# Successful Treatment of Apraxia of Eyelid Opening With Bromocriptine: A Case Report

**DOI:** 10.7759/cureus.115304

**Published:** 2026-08-27

**Authors:** Jacob Goforth, Cindy Li, Aileen Giordano

**Affiliations:** 1 Physical Medicine and Rehabilitation, University of Virginia School of Medicine, Charlottesville, USA; 2 Medical School, University of Virginia School of Medicine, Charlottesville, USA

**Keywords:** apraxia, apraxia of eyelid opening, basilar artery aneurysm, bromocriptine therapy, neurorehabilitation

## Abstract

Apraxia of eyelid opening (AEO) is a rare neurological disorder characterized by an inability to voluntarily initiate eyelid elevation despite preserved eyelid musculature and strength. Our case involved a 71-year-old female pianist who developed AEO following a ruptured basilar artery tip aneurysm complicated by right thalamic infarction, midbrain edema, intraventricular hemorrhage, and subarachnoid hemorrhage. During inpatient rehabilitation, examination demonstrated an inability to initiate the act of opening her eyes after closure despite preserved eyelid strength and the absence of blepharospasm or ptosis. This was consistent with AEO, which is primarily a clinical diagnosis. She also exhibited aphasia, limb apraxia, apraxia of speech, left hemineglect, and incomplete quadriparesis. Bromocriptine 2.5 mg nightly was initiated, off-label, to target her neglect and apraxia. Within three days, she regained the ability to voluntarily open and close her eyes. Concurrent improvements were observed in limb apraxia and apraxia of speech, allowing greater participation in rehabilitation, including resumption of piano playing. She continued to improve functionally and was ultimately discharged home with her family. Given the limited evidence supporting pharmacologic management of AEO, bromocriptine may represent a potential therapeutic option in appropriately selected patients with supranuclear eyelid opening dysfunction. Additional case series and prospective studies are needed to better define its efficacy, safety, and role in the management of AEO.

## Introduction

Apraxia of eyelid opening (AEO) is a rare condition distinguished by a patient’s inability to initiate the act of elevating the upper eyelid. While there is a relative paucity of information regarding the prevalence of the condition, one study estimated the prevalence to be 59 individuals with AEO per million individuals in the population [1]. Additionally, AEO has been found to have a two-to-one female-to-male predominance and a peak onset around the sixth decade of life [2].

Understanding the pathophysiology of AEO requires knowledge of the normal physiology of eyelid movement. The levator palpebrae is the primary muscle responsible for eyelid elevation. The orbicularis oculi is the primary muscle responsible for eyelid closure. When the eyes are open, the levator palpebrae is in its active state, and the orbicularis oculi is quiescent. In AEO, the levator palpebrae is inhibited, preventing eyelid elevation [3]. It is not due to structural abnormalities of the eyelid, muscle weakness, or fatigue. On the contrary, it is believed to stem from a disruption in higher-up neural pathways that are responsible for coordinating voluntary eyelid movement [4].

AEO is a clinical diagnosis. It is diagnosed when patients are unable to initiate the act of eyelid opening following either voluntary or involuntary lid closure. In the absence of other pathology, patients are able to maintain the eye open after it is involuntarily opened. In patients with an eyelid abnormality where AEO is suspected, other conditions that should be on the provider's differential diagnosis include conditions such as blepharospasm and ptosis. Blepharospasm is a focal dystonia characterized by involuntary contraction of the eyelids, and the provider would be assessing for abnormal twitching and involuntary closure of the eyelids. The provider would also expect to experience resistance when attempting to open the eyelids if blepharospasm were to be present. Ptosis is a condition marked by drooping of the upper eyelid, and the patient would be unable to maintain the eye fully open [3].

The case was previously presented as an oral presentation at the American Academy of Physical Medicine and Rehabilitation Annual Assembly on October 24, 2025, in Salt Lake City, Utah, United States.

## Case presentation

**Figure 1:**
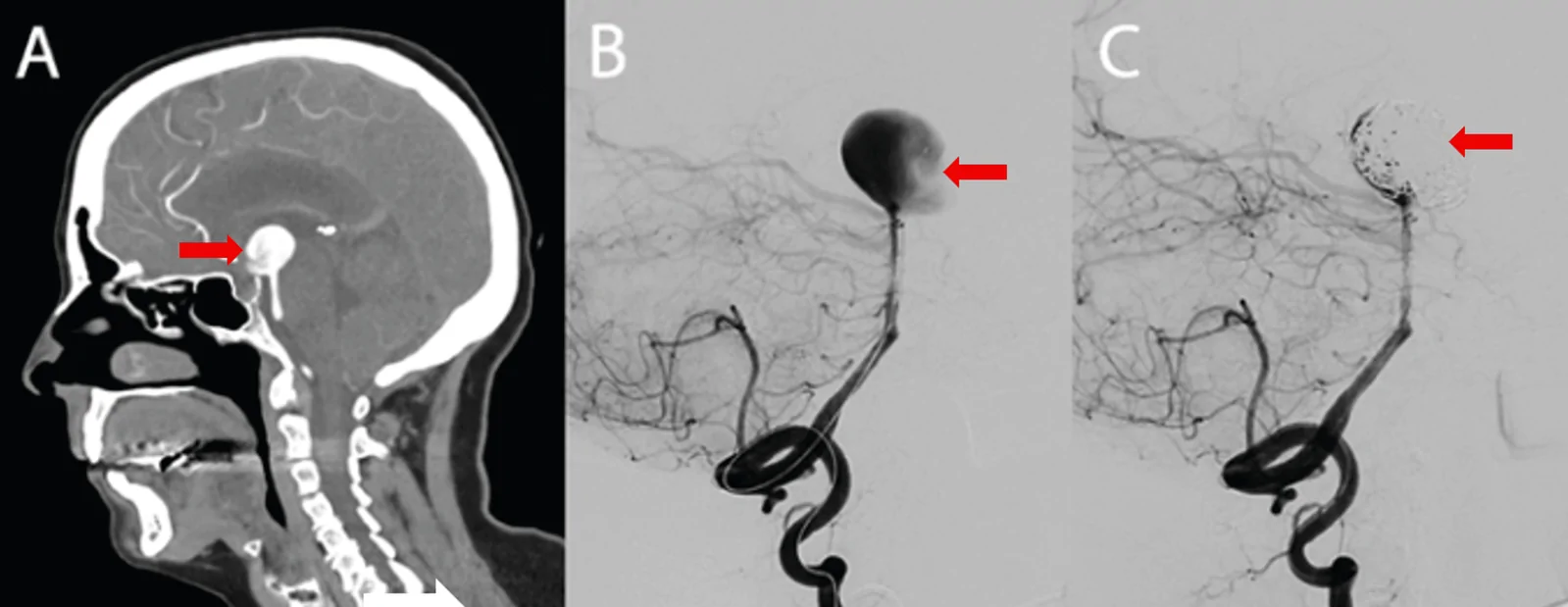
(A) CTA sagittal view showing large aneurysm of the tip of the basilar artery; (B) DSA showing large aneurysm of the tip of the basilar artery with mild contrast extravasation; (C) DSA showing basilar artery tip aneurysm status post endovascular coiling CTA: computed tomography angiography; DSA: digital subtraction angiography

**Figure 2:**
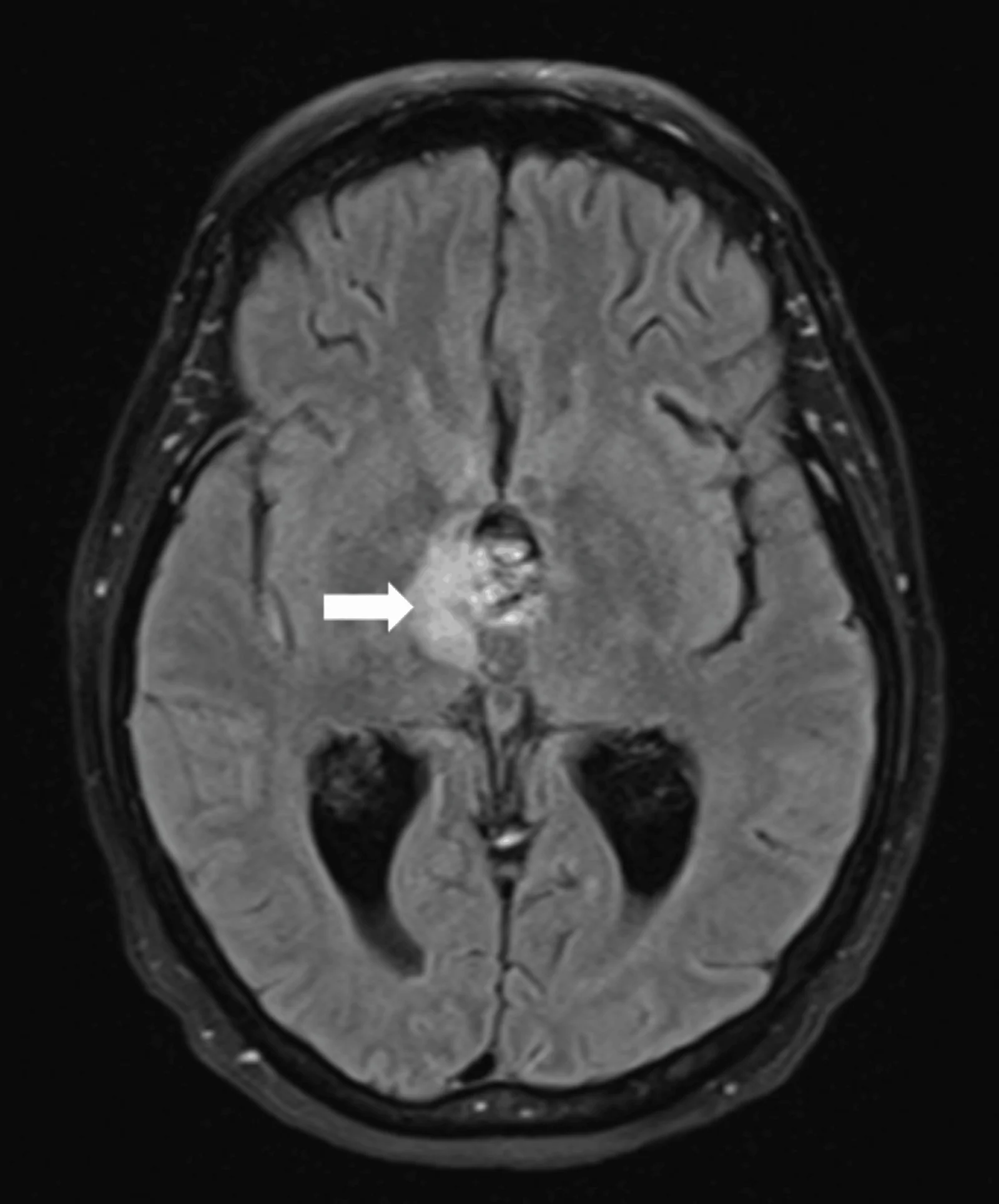
Post-embolization FLAIR (axial view) showing vasogenic edema and patchy enhancements in the right thalamus FLAIR: fluid-attenuated inversion recovery

A 71-year-old female pianist presented with a ruptured basilar artery tip aneurysm (Figure 1A). Upon return of diagnostic imaging revealing the ruptured aneurysm, the patient underwent emergent endovascular coiling (Figure 1B, 1C) and was admitted to a tertiary medical center for intensive care. Serial imaging revealed infarction of the right thalamus, significant midbrain edema, intraventricular hemorrhage, and subarachnoid hemorrhage (Figure 2).

The patient had a protracted acute care hospital initial admission, brief skilled nursing facility stay, readmission to the acute care hospital secondary to respiratory complications, and ultimately transitioned to the acute inpatient rehabilitation facility associated with the tertiary medical center approximately three months after the date of the aneurysmal rupture. 

On admission to the acute inpatient rehabilitation hospital, the patient’s physical exam was significant for incomplete quadriparesis, left hemineglect, aphasia, limb apraxia, apraxia of speech, and AEO. The diagnosis of AEO was primarily clinical and based on the following physical exam findings. The patient could not voluntarily open her eyes and kept them closed until her eyes were physically opened by the examiner. There was no abnormal contraction of the eyelids or resistance to opening, as would be expected with blepharospasm. Frontalis muscle activity was not visible on exam. After being opened by the examiner, the patient was able to keep her eyes open for an extended period, and there was no drooping of the eyelids, as would be seen in ptosis. However, following eventual voluntary closure of her eyes, she was then unable to open her eyes again on command. 

Bromocriptine 2.5 mg nightly was initiated as an off-label treatment to target the patient’s apraxia, hemineglect, and aphasia. The patient had already been trialed on Ritalin due to concern for arousal and attention deficits, but the eyelid deficits were persistent despite the Ritalin regimen. On day three of bromocriptine therapy, the patient’s eyes were found to be wide open during morning rounds. She exhibited the ability to open and close her eyes without assistance. Her apraxia of speech and limb apraxia showed improvement as well. Given that this is a single case report and that the patient was undergoing concurrent inpatient rehabilitation, it is not possible to say with absolute certainty that the bromocriptine was the sole reason that the patient experienced improvement in her eyelid opening apraxia. However, typically one would not expect this rapid rate of improvement in the patient’s AEO symptoms at three months post-injury with rehabilitation alone.

The inpatient rehabilitation therapy team subsequently set up a keyboard piano for the patient to use in the therapy gym. Upon sitting down to play the piano, she began to smile with joy as she started to play notes again. She continued to improve her mobility, activities of daily living, communication, and piano playing ability throughout her inpatient rehabilitation course. On acute inpatient rehabilitation admission, she was completely dependent with mobility and transfers. She progressed to moderate assistance with transfers and when ambulating 50 feet with two turns by her date of discharge. No adverse effects associated with bromocriptine therapy were witnessed during her time in the inpatient rehabilitation setting. She was successfully discharged home with her family.

## Discussion

AEO has been witnessed in association with extrapyramidal system pathology [5]. Injuries to the thalamus and midbrain have been associated with cases of AEO [6]. The cortical control of eyelid positioning is also thought to have a right hemispheric predominance [3]. In our patient, the thalamus and midbrain as well as the right hemisphere were injured by her aneurysm and its sequelae.

There are multiple case reports in the literature that document improvement in AEO after administration of dopaminergic agents, with most focusing on the effects of levodopa [7,8]. Multiple case reports also identify improvement in apraxia or motor function after treatment with bromocriptine [9,10]. However, this is only the second reported case, to our knowledge, that documents improvement in AEO after the initiation of bromocriptine; the first was reported by Hirayama et al. in 2000 [11].

Bromocriptine is a dopamine agonist primarily utilized for treatment of Parkinson’s disease and hyperprolactinemic disorders. Its use in apraxia and, more specifically, in AEO is off-label and relatively novel. As with any medication, it is important to consider potential side effects when initiating therapy. More common side effects of bromocriptine include nausea, vomiting, dizziness, hypotension, headache, and fatigue [12]. It is vital to monitor for these side effects and other less common side effects when initiating therapy. 

## Conclusions

AEO is a rare and very functionally impactful neurologic deficit. This patient exhibited improvement in her eyelid opening apraxia and her limb apraxia after initiation of bromocriptine. Prior literature on pharmacologic treatment for AEO is limited, likely due to the rarity of this condition, and has largely focused on levodopa. 

Based on the results of this case, bromocriptine should be considered as a potential pharmacologic option to treat AEO. Again, as with any medication, it is essential to weigh the risks and benefits and to discuss with patients and their surrogate decision makers prior to initiating pharmacotherapy. While bromocriptine was associated with resolution of AEO for this patient, its use for this indication is considered to be relatively novel. Further studies of bromocriptine use in the setting of AEO, including case series and randomized controlled trials, are needed to more robustly validate bromocriptine’s efficacy in treating AEO.
